# Prevalence of *Borrelia burgdorferi* Sensu Lato in Ticks from Eastern China

**DOI:** 10.4269/ajtmh.14-0587

**Published:** 2015-02-04

**Authors:** Juan Hou, Feng Ling, Chengliang Chai, Ye Lu, Xianghua Yu, Junfen Lin, Jimin Sun, Yue Chang, Xiaodong Ye, Shiping Gu, Weilong Pang, Chengwei Wang, Xiaohua Zheng, Jianmin Jiang, Zhiping Chen, Zhenyu Gong

**Affiliations:** Zhejiang Provincial Center for Disease Control and Prevention, Hangzhou, China; Wenzhou Municipal Center for Disease Control and Prevention, Wenzhou, China; Taizhou Municipal Center for Disease Control and Prevention, Taizhou, China; Jindong Center for Disease Control and Prevention, Jindong, China; Anji Center for Disease Control and Prevention, Anji, China; Tiantai Center for Disease Control and Prevention, Tiantai, China; Daishan Center for Disease Control and Prevention, Daishan, China; Xianju Center for Disease Control and Prevention, Xianju, China

## Abstract

To explore the tick distribution and prevalence of *Borrelia* in Zhejiang Province, we performed a survey in nine sites. A total of 447 adult ticks of 11 species were captured and the dominant tick species were *Haemaphysalis longicornis* and *Ixodes sinensis* and the abundance of tick species in different areas varied significantly. Overall, 4.70% of the ticks were polymerase chain reaction (PCR) positive for *Borrelia*. The average PCR positive rates were 5.19% for *H. longicornis*, 3.45% for *Amblyomma testudinarium*, 1.06% for *I. sinensis*, 5.00% for *Rhipicephalus* (*Boophilus*) *microplus*, and 19.44% for *Ixodes granulatus*, respectively. No *Borrelia* DNA was detected in *Rhiphicephalus haemaphysaloides*, *Haemaphysalis yeni*, *Dermacentor taiwanensis*, *Haemaphysalis hystricis*, *Hyalomna asiaticum*, and *Ixodes ovatus*. The prevalence of *Borrelia* was significantly different among tick species and the prevalence in *I. granulatus* was significantly higher than that in other tick species. Of note, experimentally confirmed vectors for *B. burgdorferi* s.l. including *I. sinensis* and *I. granulatus* were found in Zhejiang Province. Two species of *B. burgdorferi* s.l. exist in Zhejiang Province of which 12 sequences were most similar to the sequence of *Borrelia garinii* and nine sequences were most similar to the sequence of *Borrelia valaisiana* or *Borrelia yangtze* sp. nov.

## Introduction

Ticks are important vectors for human and animal pathogens of viral, bacterial, and protozoan nature worldwide. Lyme disease is a tick-borne disease that is common in all temperate regions of the Northern Hemisphere and it is the most common tick-borne disease in the United States. Most infections occur during the months of May through August, when both the nymph ticks' activity and human outdoor activity are at their peak. The causative agent for Lyme disease, *Borrelia burgdorferi* s.l., was first identified from the hard tick *Ixodes scapularis* (formerly called *I. dammini*) in the autumn of 1981.^1^,^2^ To date, at least 19 species of *Borellia burgdorferi* s.l. have been described: *B. burgdorferi* sensu stricto, *B. garinii*, *B. afzelii*, *B. japonica*, *B. valaisiana*, *B. lusitaniae*, *B. andersonii*, *B. tanukii*, *B. turdi*, *B. bissettii*, *B. sinica*, *B. spielmanii*, *B. californiensis*, *B. carolinensis* sp. nov., *B. americana*, *B. bavariensis*, *B. finlandensis*, *B. kurtenbachii*, and *B. yangzte*.^3^–^13^

In China, the first case of Lyme disease was reported in Hailin county Heilongjiang province in 1987.^14^ Thereafter, Lyme disease has been documented in more than 20 provinces and autonomous regions throughout China and several *B. burgdorferi* s.l. genotyping studies have been conducted.^8^,^15^–^18^ Some previous reports have shown the presence of antibody immunoglobulin G (IgG) against *B. burgdorferi* s.l. in serum of humans from different areas in Zhejiang Province.^19^–^21^ Monitoring tick distribution and the prevalence of *Borrelia* are essential to describe and understand the risk of acquiring *Borrelia* infections. To explore the tick distribution and prevalence of *Borrelia*, we performed a survey in nine locations in Zhejiang Province in Eastern China.

## Materials and methods

### Tick sampling.

**Figure 1.**
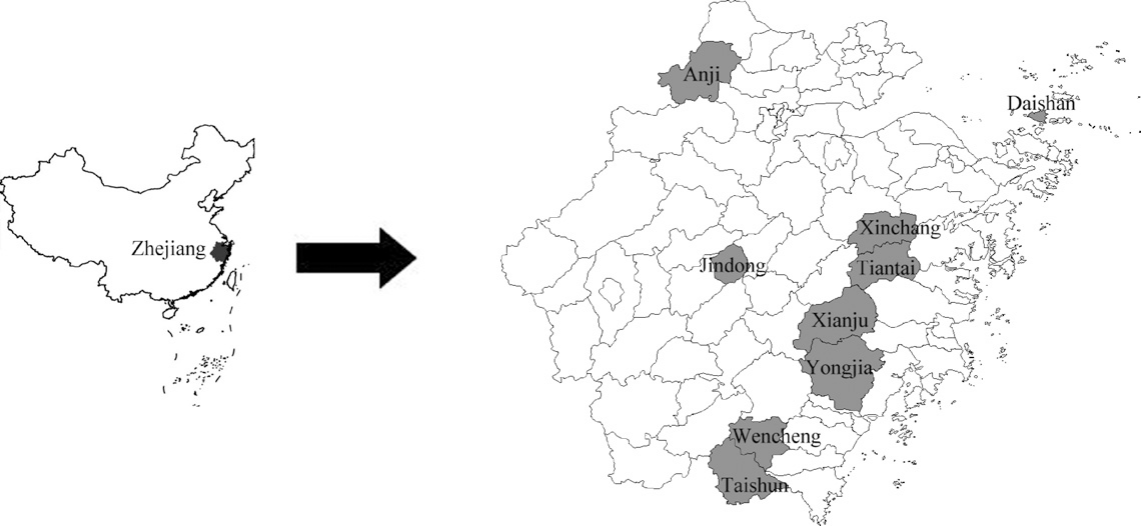
Geographical distribution of investigated sites in Zhejiang Province.

The investigated sites included Daishan, Xinchang, Jindong, Tiantai, Xianju Yongjia, Wencheng, Taishun, and Anji and were randomly chosen based on their geographical and administrative locations (Figure 1). Some ticks were collected from domestic animals such as sheep, cattle, and dog using tweezers with the help of the owners of these domestic animals. Some ticks were collected from *Sus scrofa* trapped by local hunters or sold at markets. The other ticks were collected from wild mammals including *Erinaceus amurensis*, *Apodemus agrarius*, *Rattus niviventer*, and *Suncus murinus*, which were trapped with mousetraps. Collections of tick samples were conducted during January 2010 to December 2011. Only adult ticks were collected and no questing ticks were collected. All ticks were identified to the species level by standard guides and were stored at −20°C before DNA extraction.

### DNA extraction.

Each adult tick was subjected individually to DNA extraction. Ticks were washed using 70% ethanol once; they were then washed three times with sterile deionized water to decontaminate the surface. Individual ticks were placed into different sterilized mortars and crushed with corresponding sterile pestles with liquid nitrogen. The DNA was prepared from the crushed ticks using the QIAamp Tissue Kit (Qiagen, Hilden, Germany) according to the manufacturer's instructions.

### Polymerase chain reaction (PCR) amplification.

All tick samples were screened for *Borrelia* infection through testing them individually by nested PCR amplification, which was performed using a MyCyclerThermal Cycler (BIO-RAD, Hercules, CA) with the primers (Table 1) designed to amplify a fragment of 5S-23S rRNA as described previously.^6^

The reaction mixtures contained 10 mM Tris-HCl, 1.5 mM MgCl2, 50 mM KCl (pH 8.3), 200 mM each dNTP, 1.25 U Taq polymerase, and 0.5 mM each respective primer. Nested PCR was performed using 1 uL of the primary PCR product as the template. The PCR products were run in a 1.5% agarose gel, stained with gold view, and visualized using UV light. To avoid cross-contamination, all steps were performed in separate rooms; mastermix was prepared under a laminar air flow bench. In each PCR, at least two negative controls contained mastermix and sterile water instead of DNA template.

### Cloning and sequencing of PCR products.

After electrophoresis, all positive DNA amplicons were purified using the Promega Wizard PCR Preps Kit (Promega, Madison, WI) and then cloned into the PGEM-T Easy vector system (Promega) following the manufacturer's protocol. The white recombinant clones were selected for sequencing. Bidirectional sequencing of positive PCR products were commercially conducted by Shanghai Sangon Biotechnology Co. (Shanghai, China).

### Database DNA comparisons.

Our sequences were compared with published sequences using the Basic Local Alignment Tool (BLAST) program from the National Center for Biotechnology Information Website (http://blast.ncbi.nlm.nih.gov/Blast.cgi).

### Data analysis.

Logistic regression analysis, χ^2^ test, or Fisher's exact test was used to compare *Borrelia* prevalence among different tick species, different sampling sites, and different host species. The dependent variable in the logistic regression was assigned as the prevalence status and the independent variables were tick species, sampling site, host species, tick species × sampling site, tick species × host species, sampling site × host species. The method of logistic regression used was forward-conditional. The stepwise probability was set to 0.05 for entry and 0.10 for removal. The classification cut-off was 0.5 and the maximum number of iterations was 20. Omnibus tests of model coefficients were also conducted. The difference was considered statistically significant when *P* < 0.05. Statistical analysis was performed with the use of Statistical Product and Service Solutions (SPSS 11.0; Chicago, IL).

## Results

### Tick samples.

A total of 447 adult ticks of 11 species were captured (Table 2). The dominant tick species were *Haemaphysalis longicornis* (47.43%) and *Ixodes sinensis* (21.03%) in Zhejiang Province. However, the abundance of tick species varied significantly (X2 = 1071.681, *P* = 0.000 < 0.05) in different areas. *Haemaphysalis longicornis* was dominant in Daishan (91.38%), Xinchang (80.00%), Taishun (76.67%), and Yongjia (94.29%); *H. longicornis* (21.43%), *Rhiphicephalus haemaphysaloides* (21.43%), and *I. sinensis* (22.86%) in Jindong; *I. sinensis* (100%) in Tiantai; *H. longicornis* (58.14%) and *Rhipicephalus microplus* (41.86%) in Xianju; *Amblyomma testudinarium* (23.96%) and *I. sinensis* (65.63%) in Anji; *Ixodes granulatus* (100%) in Wencheng (Table 2). *Haemaphysalis longicornis* ticks were collected from sheep (38.58%), cattle (54.72%), and *Sus scrofa* (6.60%). *Ixodes sinensis* ticks were from sheep (63.83%) and cattle (36.17%). *Ixodes granulatus* ticks were from *Apodemus agrarius* (41.67%), *Rattus niviventer* (27.78%), and *Suncus murinus* (30.55%). Additionally, *R. haemaphysaloides* and *A. testudinarium* were all from domestic dogs, all *B. microplus* were from cattle, *H. yeni*, *H. hystricis*, and *H. asiaticum* from *S. scrofa*, *D. taiwanensis* and *I. ovatus* were from *Eragrotis amurensis*.

### Prevalence of *Borrelia* infection.

Overall, 4.70% (21 of 447) of the ticks were PCR positive. The average PCR-positive rates were 5.19% (11 of 212) for *H. longicornis*, 3.45% (1 of 29) for *A. testudinarium*, 1.06% (1 of 94) for *I. sinensis*, 5.00% (1 of 20) for *R. microplus*, and 19.44% (7 of 36) for *I. granulatus*, respectively (Table 2). No *Borrelia* DNA was detected in *R. haemaphysaloides*, *H. yeni*, *D. taiwanensis*, *H. hystricis*, *H. asiaticum*, and *I. ovatus*. The prevalence of *Borrelia* varied significantly among species (χ^2^ = 24.106, *P* = 0.012 < 0.05) and the prevalence in *I. granulatus* was significantly higher than that in other species of ticks (*P* = 0.001, Table 2).

The prevalences in Daishan, Xinchang, Jindong, Tiantai, Xianju, Anji, Wencheng, Taishun, and Yongjia were 0, 0, 2.86%, 6.67%, 23.26%, 1.04%, 23.33%, 0, and 0, respectively (Table 2). The prevalences of Xianju and Wencheng were significantly higher than that of other areas (χ^2^ = 69.385, *P* = 0.000 < 0.05).

According to results of logistic analysis, the χ^2^ value in omnibus tests of model coefficients was determined as 28.835 (*P* = 0.000). Furthermore, the overall correct percentage was found to be 95.3%. Variables in the equation below included tick species and sampling site×host species and place of residence and the Wald values were determined to be 11.465 (*P* = 0.001) and 16.443 (*P* = 0.000). The equation was

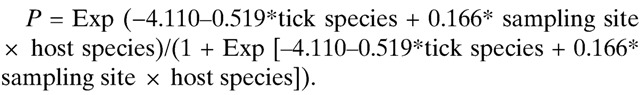


### Comparison to the sequences in the GenBank.

As shown in Table 3, 12 sequences were 99–100% identical to three *B. garinii* (GenBank accession nos.: L30119, AB178361, AB091797, AF497990), which were all *B. garinii* 5S-23S rRNA partial sequences detected from *I. ricinus* in Russia, Turkey, and Czech Republic, respectively. The sequences of samples 145, 168, 344, and 345 were identical to *B. valaisiana* 5S-23S rRNA partial sequence (GenBank accession no. HM100120), which was detected in *I. granulatus* ticks from Taiwan. Moreover, the sequences of samples 349, 350 were identical to another *B. valaisiana* 5S-23S rRNA partial sequence (GenBank accession no. HM100125), which was detected in *I. granulatus* ticks from Taiwan and the sequence of 354 had the highest sequence similarity (99%) to this sequence. The sequence of sample 348 was most similar (96%) to *B. valaisiana* 5S-23S rRNA partial sequence (GenBank accession no. JX888445), which was detected from human blood in Heilongjiang Province, China. The sequence of sample 360 was identical to *B. yantze* strain QSYSP3 5S-23S ribosomal RNA partial sequence detected in *H. longicornis* from southwestern China.

All sequences that were most similar to *B. garinii* were detected in *H. longicornis* except for one sequence that was detected in *A. testudinarium* (KJ398184). The majority of sequences that were most similar to *B. valaisiana* were detected in *I. granulatus* except for two sequences, one sequence was from *I. sinensis* and the other was from *B. microplus* (Table 3). The sequences that were most similar to *B. garinii* came from three areas (Jindong, Xianju, Anji) and the sequences that were most similar to *B. valaisiana* came from three additional areas (Tiantai, Xianju, Yongjia). Moreover, *H. longicornis* and *A. testudinarium* ticks, *Borreia* sequences detected in them were most similar to *B. garinii,* were collected from cattle and dogs, respectively. *Ixodes granulatus*, *I. sinensis*, and *B. microplus*, *Borrelia* sequences detected in them were most similar to *B. valaisiana,* were collected from *S. scrofa*, cattle, *A. agrarius*, and *R. niviventer*.

## Discussion

Previous studies in China reported the prevalence and genetic characteristics of *B. burgdorferi* s.l. in ticks collected from different sites in Eastern China. A total of 11 species of ticks were found in nine areas of Eastern China. The distribution of tick species varied considerably in the different areas. This variability may have been caused by different hosts from which ticks were collected and different habitats in sampling sites. The dominant tick species of Zhejiang Province are *H. longicornis* and *I. sinensis*.

In our study, the overall infection rate with *B. burgdorferi* s.l. was found to be 4.70% among ticks as detected using nested PCR. Previous studies reported that *Borrelia* spp. were detected in 22 of 55 (40.00%) ticks,^17^ 41 of 182 (22.53%) ticks,^22^ 94 of 667 (14.09%) ticks,^23^ 25.6% of *I. persulcatus* ticks,^24^ 123 of 181 (67.96%) *I. granulatus* ticks,^25^ 27 of 147 (18.37%) ticks,^26^ and 23 of 113 (20.35%) ticks.^27^ The lower infection rate in our study is most probably a result of different sampling regions, different tick species, different methods of tick collection or the differences in sensitivity for the different methods used for *Borrelia* detection.

Our data showed that the prevalences in *I. granulatus* and *H. longicornis* were higher than that in other species of ticks. The findings indicate that *I. granulatus* and *H. longicornis* may play roles as vector or carrier of *Borrelia* in Eastern China. However, we cannot exclude that other ticks might act as carriers for *B. burgdorferi* s.l. because of small sample sizes. Additionally, former studies have shown that *I. granulatus* ticks are vector of *Borrelia*, but *H. longicornis* only carry *Borrelia* and cannot transmit it.^28^,^29^ Therefore, *H. longicornis* ticks are not the main vector of *Borrelia*, although they were dominant in Zhejiang Province. Similarly, *I. sinensis* ticks were experimentally confirmed vector and *H. yeni* ticks were confirmed as being incapable of serving as a vector for *B. burgdorferi* s.l. Other tick species collected in our study have unknown vector status (Table 2). Of *B. burgdorferi* s.l. complex spirochetes, *B. afzelii*, *B. bavariensis*, *B. garinii*, *B. spielmanii* and *B. burgdorferi* sensu stricto are known to be pathogenic for humans, and *B. valaisiana*, and *B. lusitaniae* are considered potentially pathogenic.^30^ The finding of *B. garinii* and *B. valaisiana* or *B. yangtze* sp. nov. in ticks that were LD vectors indicate that transmission of LD via tick bite in Zhejiang Province is possible.

A report on distribution of *B. burgdorferi* s.l. in China shows that *B. garinii* was the main genotype in China and distributed mainly in northern China, *B. afzelii* was the second most frequently found species and distributed in both northern and southern China, and all *B. valaisiana* strains were isolated from southern China.^31^ Our study confirmed that two species of *B. burgdorferi* s.l. might exist in Zhejiang Province of which 12 sequences were most similar to the sequence of *B. garinii* and the other sequences were most similar to the sequence of *B. valaisiana* or *B. yangtze* sp. nov. The reason might be that Zhejiang Province is located in east-central China. The sequences, which were found in our study, are similar to sequences that have been designated *B. yangtze* sp. nov. or *B. valaisiana* in different studies. To determine the proper species designation further studies are required using multilocus sequence typing.

The majority of sequences that were most similar to *B. garinii* were detected in *H. longicornis* and the majority of sequences that were most similar to *B. valaisiana* or *B. yangtze* sp. nov. were detected in *I. granulatus* suggesting a relation between tick species and *Borrelia* species. Furthermore, *B. garinii* and *B. valaisiana* were detected from ticks collected from different host species indicating a relation between host species and *Borrelia* species. Cattle and dogs might be reservoirs for *B. garinii* and *S.scrofa,* cattle, *A. agrarius*, and *R. niviventer* might be reservoirs for *B. valaisiana.* The difference in *Borrelia* species distribution may be also relative to different tick species. *Borrelia garinii* is considered to be transmitted by birds, but hosts from which ticks were collected did not include birds in our study. As all ticks were adult, it is likely that *Borrelia* infections of these ticks were acquired during a previous blood meal.

There were several limitations to our study. First, the low numbers of ticks of each species in each region collected in different times and from different animals reduced the probabilities of getting useful information from this study. Second, the examined ticks were collected from animals rather than as questing specimens, which brings into question whether *B. burgdorferi* s.l. spirochetes would survive the molt, especially for non-*Ixodes* ticks. Finally, tick species from different sites varied significantly, which might influence infection rates of different sites.

In conclusion, the dominant tick species were *H. longicornis* and *I. sinensis* in Zhejiang Province. We detected *B. burgdorferi* s.l. in diverse species of ticks from different areas. *Borrelia burgdorferi* s.l. detected were similar to *B. garinii*, *B. valaisiana*, or *B. yangtze* sp. nov*.*, which can cause human infections, indicating that *B. burgdorferi* s.l. infections might be largely unrecognized and these infections may be frequent in Zhejiang Province.

## Figures and Tables

**Table 1:** Polymerase chain reaction primers

Primer name	Target gene	Primer sequence (5′-3′)	Anneal temperature	Product size (bp)
B1	5S-23S rRNA	CGACCTTCTTCGCCTTAAAGC	55°C	
B2		TAAGCTGACTAATACTAATTACCC		
B3		TCCTAGGCATTCACCATA	59°C	245
B4		CTGCGAGTTCGCGGGAGA		

**Table 2:** Prevalence of *Borrelia* infection among different species ticks from different areas

	Dai shan	Xin chang	Jin dong	Tian tai	Xian ju	An ji	Wen cheng	Tai shun	Yong jia	Total (n)	No. PCR positive (n)	Prevalence (%)
*Haemaphysalis longicornis^−^*	53	8	15	0	25	9	0	69	33	212	11	5.19
*Rhipicephalus haemaphysaloides*^?^*^−^*	3	2	15	0	0	0	0	0	0	20	0	0
*Amblyomma testudinarium*^?+^	0	0	5	0	0	23	0	1	0	29	1	3.45
*Ixodes sinensis*^+^	0	0	16	15	0	63	0	0	0	94	1	1.06
*Boophilus microplus*^?+^	1	0	0	0	18	0	0	0	1	20	1	5.00
*Haemaphysalis yeni*^−^	0	0	5	0	0	0	0	14	0	19	0	0
*Dermacentor taiwanensis*^?−^	0	0	9	0	0	0	0	0	0	9	0	0
*Haemaphysalis hystricis*^?−^	0	0	5	0	0	0	0	0	0	5	0	0
*Hyalomma asiaticum*^?−^	0	0	0	0	0	1	0	0	0	1	0	0
*Ixodes granulates*^+^	1	0	0	0	0	0	30	4	1	36	7	19.44
*Ixodes ovatus*^?+^	0	0	0	0	0	0	0	2	0	2	0	0
Total (n)	58	10	70	15	43	96	30	90	35	447	21	4.70
Positive (n)	0	0	2	1	10	1	7	0	0			
Prevalence (%)	0	0	2.86	6.67	23.26	1.04	23.33	0	0			

Experimentally confirmed vector (+), experimentally confirmed as being incapable of serving as a vector (−), unknown vector status but likely to be a vector (?+), unknown vector status but not likely to be a vector (?−).

**Table 3:** Sequence names, GenBank accession number, similar species, and source

Sequence names and accession nos.	Similar species	Source	Location
78 (KJ398171), 80 (KJ398172)	*B. garinii* (strain 20047)	*H. longicornis*	Jindong
182 (KJ398175), 186 (KJ398176), 187 (KJ398177), 188 (KJ398178),	*B. garinii* (strain 20047)	*H. longicornis*	Xianju
189 (KJ398179), 190 (KJ398180), 191 (KJ398181), 194 (KJ398182),			
195 (KJ398183)			
199 (KJ398184)	*B. garinii* (strain 20047)	*A. testudinarium*	Anji
145 (KJ398173)	*B. valaisiana* (strain KM36)	*I. sinensis*	Tiantai
168 (KJ398174)	*B. valaisiana* (strain KM36)	*B. microplus*	Xianju
344 (KJ398185), 345 (KJ398186), 348 (KJ398187), 349 (KJ398188),	*B. valaisiana* (strain KM36, strain HL8),	*I. granulatus*	Yongjia
350 (KJ398189), 354 (KJ398190), 360 (KJ398191)	*B. yantze* (strain QSYSP3)		
